# Feasibility and acceptability of an evidence-informed digital intervention to support self-management in people with non-alcoholic fatty liver disease: protocol for a non-randomised feasibility study (VITALISE)

**DOI:** 10.1186/s40814-023-01286-2

**Published:** 2023-04-19

**Authors:** Leah Avery, Hollie Smith, Stuart McPherson, Kate Hallsworth

**Affiliations:** 1 School of Health & Life Sciences, Teesside University, Tees Valley, Middlesbrough, UK; 2 Translational and Clinical Research Institute, Newcastle University, Newcastle Upon Tyne, UK; 3 Liver Unit, Newcastle Upon Tyne Hospitals NHS Foundation Trust, Newcastle Upon Tyne, UK; 4 NIHR Newcastle Biomedical Research Centre, Newcastle Upon Tyne, UK

**Keywords:** Non-alcoholic fatty liver disease, NAFLD, Lifestyle behaviour change, Diet, Physical activity, Weight loss, Supported self-management, Digital intervention, Feasibility study

## Abstract

**Background:**

Non-alcoholic fatty liver disease (NAFLD) represents a spectrum of disease ranging from simple fatty liver to non-alcoholic steatohepatitis, cirrhosis, liver cancer and liver failure. NAFLD affects up to 30–40% of adults in Western countries and is directly linked to overweight and obesity. There are no approved drugs to specifically target NAFLD, therefore weight loss achieved through changes in dietary and physical activity behaviours is the recommended management approach. However, achieving and sustaining weight loss is challenging for patients with NAFLD. We developed a NAFLD-specific digital lifestyle intervention (VITALISE) to target changes in dietary and physical activity behaviours of patients with NAFLD to initiate weight loss and weight loss maintenance. This study aims to evaluate the feasibility and acceptability of VITALISE in a secondary care clinical setting.

**Methods:**

A single-centre, one-arm, prospective design will be used to assess the feasibility and acceptability of recruitment, uptake, engagement and completion of VITALISE. Health-related outcomes will be assessed at baseline and 6-months. An interim measure of self-reported weight, physical activity and self-efficacy will be recorded at 12-weeks. Qualitative semi-structured interviews conducted at 6 months follow up will further explore acceptability and feasibility and fidelity of receipt and enactment. The study aims to recruit 35 patients with newly diagnosed NAFLD over a 6-month time period. Eligible patients will have continuous access to VITALISE and monthly tele-coaching support for 6 months prior to follow-up with a hepatologist.

**Discussion:**

VITALISE offers access to evidence and theory-informed tailored dietary and physical activity support for patients with NAFLD. The intervention is designed for use by patients in their own time, outside of the hospital setting to overcome well documented challenges including attending additional appointments, and lack of time during routine appointments to adequately address lifestyle behaviour change. This feasibility study will determine the feasibility of VITALISE to support clinical care delivery.

**Trial registration:**

ISRCTN12893503.

## Introduction

Non-alcoholic fatty liver disease (NAFLD) affects up to 30–40% of adults in Western countries and is the most common liver condition worldwide [1, 2]. It represents a spectrum of liver disease ranging from simple fatty liver through to non-alcoholic steatohepatitis (NASH – liver inflammation), life threatening cirrhosis, liver cancer and liver failure, and has become a common cause of liver transplant. Approximately 40% of patients with NAFLD will develop progressive liver fibrosis and ultimately, 5–11% will develop end-stage liver disease [3, 4].

NAFLD is directly linked to being overweight or obese, usually caused by chronic excess calorie consumption and a lack of physical activity and exercise [5]. Currently, there are no approved drugs to specifically target or treat NAFLD. Weight loss, achieved through change in dietary and physical activity behaviours is the recommended treatment for NAFLD, which can reduce liver fat, inflammation and fibrosis [6, 7]. Evidence-based clinical guidelines for the management of NAFLD state the importance of lifestyle behaviour change in patients with NAFLD, regardless of disease severity [8–10]. However, people with NAFLD find achieving sustained weight loss a significant challenge, and clinicians struggle to support patients with NAFLD due to a lack of specific training, interventions, and referral pathways [11–13].

We systematically developed an evidence and theory-informed NAFLD-specific digital lifestyle intervention using Intervention Mapping [14]—VITALISE (inter**V**ention to promote l**I**fes**T**yle change in non-**A**lcoholic fatty **LI**ver disea**SE**) [15]. Intervention Mapping provides a systematic approach to developing theory- and evidence-informed interventions that integrates the requirements of the target population [14]. It provides a step-by-step account of the translation of theory- and evidence-based behaviour change techniques into intervention components, which explicitly target theoretical determinants of behaviour and behaviour change [14]. This process enables replication in terms of development and delivery of interventions and facilitates intervention optimisation and robust evaluation. VITALISE was co-designed with patients and healthcare professionals (HCPs) and addresses the pressing need for a behaviour change programme for people with NAFLD, targeting dietary and physical activity behaviours to initiate weight loss and weight loss maintenance. It provides guideline-recommended structured education and self-regulation tools to facilitate supported self-management. Together these enable patients to plan and track changes, and develop strategies to overcome any barriers encountered, supported by lifestyle coaches.

## Aims and objectives

The aim of the study is to evaluate the feasibility and acceptability of VITALISE in the clinical setting within secondary care. This feasibility study will not include formal hypothesis testing because it is not powered to detect clinically meaningful changes in outcomes. The main aim is to gain insight into whether patients find VITALISE acceptable and whether it is feasible to deliver within routine clinical care.

In accordance with NIHR and CONSORT guidance for feasibility studies [16, 17], the primary objectives are to determine recruitment, retention and attrition rates, intervention uptake, engagement, adherence, and follow-up rates and to test the robustness of our data collection procedures. We will collect data on usability and individual patient views on VITALISE via semi-structured interviews to add important context to quantitative outcome data. Importantly, we will look at the demographics of patients who participate versus those who decline. We will seek information from those who decline to explore ways in which these barriers to participation can be overcome.

The secondary objective is to determine whether participation in the intervention leads to changes in outcomes including physical activity, diet, body weight, HbA1c, liver enzymes, liver stiffness, blood pressure, lipid profile (all measured as part of standard care using the NAFLD Care Bundle) [18] and patient activation/empowerment. These preliminary data will provide some indication of whether the intervention could be effective as part of routine clinical care.

## Methods

### Study design

This feasibility study will use a single-centre (Newcastle upon Tyne Hospitals NHS Foundation Trust (NuTH)), one-arm, prospective design. Feasibility and acceptability of recruitment, the intervention (VITALISE), and associated outcome measures will be assessed using a mixed-methods approach. Health-related outcomes will be quantitatively assessed at baseline and 6-months. An interim measure of self-reported weight, physical activity and self-efficacy (via the Patient Activation Measure (PAM)) will be recorded at 12-weeks.

### Sample size

With reference to best practice guidelines on sample size requirements for feasibility studies [19] and practical time frames, we will aim to recruit 35 patients over a 6-month time period.

### Target population

Adults with newly diagnosed NAFLD (see inclusion criteria for definition).

### Patient identification and consent process

Patients will be recruited from the Liver Unit at NuTH. Eligible patients will be given/sent a study invitation letter and participation information sheet. At the clinic visit, the treating physician will discuss the study with eligible patients following receipt of standard NHS care (i.e. review, examination, and blood tests). If the patient is interested in taking part, a research team member will provide them with more information about the study, answer any questions, obtain written informed consent, collect baseline data, and offer an induction to the intervention (either on the same day as the clinic appointment, or on an alternative day if preferred by the patient).

#### Inclusion criteria

• Aged over 18 years
• Clinical diagnosis of NAFLD following review by a hepatologist within the last 6-months
• Willing and able to provide written informed consent

#### Exclusion criteria

• Decompensated NASH cirrhosis (Child Pugh score ≥ 7)
• Diagnosed/previous eating disorder or purging as recorded in the medical notes
• Potentially harmful alcohol consumption (>14 units/week for females; >21 units/week for males)
• Known cancer (except skin cancer)
• Myocardial infarction within 6 months or uncontrolled cardiovascular disease
• Pregnant/considering pregnancy
• Inability to read and converse in English

### Preparation procedures

A member of the research team will use a study-specific medical questionnaire to collect sociodemographic and clinical data including information on medical history and current medications (See supplementary file). Body weight, height, waist and hip circumference, blood pressure, blood tests (glucose, lipid profile, liver blood tests [LBTs], full blood count [FBC], HbA1c), liver stiffness measurement [LSM] by Fibroscan and calculation of clinical scores (QRISK^3^, Fibrosis-4 (FIB-4) Score, NAFLD Fibrosis Score (NFS)) will be measured as part of standard care. Patients with Type 2 diabetes (T2DM) will be monitored as part of standard care and medications adjusted in response to weight loss/dietary change/physical activity as necessary. This will be reported as part of the evaluation.

Patients will be asked to complete a physical activity questionnaire (the Godin leisure-time physical activity questionnaire [20]) and Patient Activation Measure (PAM) [21] as part of the research study at baseline, 12-weeks and 6-month follow-up. Patients will also be given an accelerometer to wear for 7-days (to objectively measure physical activity levels) which will be returned to the research team in a pre-paid envelope at baseline and 6-month follow-up.

VITALISE has been developed in collaboration with a commercial organisation, ‘Changing Health Limited’, who are a leading provider of lifestyle behaviour change programmes to the NHS (Changing Health | Digital Behaviour Change At Scale). Changing Health provide evidence-based education and lifestyle coaching digital solutions for people with type 2 diabetes across the UK and renders its innovative services customised to their clients’ requirements. With the patient’s permission, the research team will send their email address, and full name to Changing Health who will subsequently email the patient a welcome email allowing them to access the VITALISE intervention and setup their profile. A researcher will provide patients with a demonstration of how to login to VITALISE. Table 1 outlines the summary schedule of investigations.

**Table 1:** Summary schedule of investigations: (* = completed as part of standard care). Patients will be asked to self-report their weight, physical activity levels and complete the PAM at 12-weeks. This interim “visit” will be conducted by telephone

Visit	1	2 (Virtual)	3
**Timescale**	**0**	**12-weeks**	**6-months**
**Study Procedure**			
Review inclusion/exclusion	X		
Obtain informed consent	X		
Height*	X		
Weight*	X	X	X
Waist and hip circumference*	X		X
Blood pressure*	X		X
Fibroscan* (LSM^1^, IQR/med ratio^2^, CAP^3^ and CAP IQR^4^)	X		X
Bloods: Glucose*, Lipid profile*, LBTs^5^*, FBC^6^*, HbA1c^7^*	X		X
Calculation of: QRISK3^8^*, FIB-4^9^*, NFS^10^*	X		X
Physical activity: Step count Accelerometry (7-days)	X X	X	X X
Questionnaires: PAM Score^11^, Godin Physical Activity	X	X	X
Semi-structured interview			X

^1^Liver stiffness measurement

^2^Interquartile range/median ratio

^3^Controlled attenuation parameter

^4^Controlled attenuation parameter interquartile range

^5^Liver blood tests

^6^Full blood count

^7^Glycated haemoglobin

^8^Cardiovascular disease risk score

^9^Fibrosis-4 Score

^10^NAFLD Fibrosis Score

^11^Patient Activation Measure

### Intervention

Patients will be given a personal login for VITALISE and will have continuous access for a 6-month period prior to their follow-up appointment with a hepatologist. They will be supported by monthly personalised tele-coaching sessions delivered by specifically trained lifestyle coaches. A pedometer will be provided to allow patients to record daily step count. Table 2 provides and overview of VITALISE content.

**Table 2:** Overview of VITALISE content: to be added into page 9 under table 2 heading

Module	Sub-components	Description of information content
**Module 1: Introduction**	**Welcome to VITALISE with Changing Health**	Message from a specialist in lifestyle management of NAFLD providing an overview of the aims and objectives of the programme
**Module 2: Understanding non-alcoholic fatty liver disease (NAFLD)**	**What is NAFLD?**	Overview of the liver; what is NAFLD and how it progresses; the different stages of the disease
	**How is NAFLD diagnosed?**	Overview of the tests/investigations that may be used to diagnose NAFLD
	**Understanding your health results**	What the different tests/investigations measure and what the results mean
	**NAFLD myths**	Common myths about NAFLD and why it shouldn’t be left unmanaged
	**Managing your NAFLD**	Animations demonstrating how lifestyle change can improve liver health, reduce cardiovascular disease risk and improve metabolic control
	**Patient success stories**	Real-life examples of how people with NAFLD or at risk of NAFLD have improved their liver health by making lifestyle changes
	**Long term heath complications from NAFLD**	Potential consequences of not managing NAFLD
**Module 3: Getting started with coaching**		How to access lifestyle coaches and what to expect from coaching sessions
**Module 4: Food and NAFLD**	**Energy balance**	How to balance energy requirements in the context of weight loss
	**Nutrition and NAFLD**	The relationship between diet quality, eating patterns and NAFLD
	**Understanding carbohydrates**	What are carbohydrates and the role they play in our diet
	**Understanding fats**	What are fats and the role they play in our diet
	**Understanding alcohol**	Recommended alcohol intake; calorie content in alcoholic drinks; provision of an online alcohol calculator
**Module 5: How do I make changes to my diet?**	**Finding the right dietary approach**	Animation explaining the evidence supporting two dietary approaches for managing NAFLD
	**Calorie restriction**	Calorie restriction in more detail; provision of online weight loss calculators and calorie checkers; link to the NHS weight loss plan
	**The Mediterranean diet**	Mediterranean diet in more detail
	**Other dietary approaches**	Information and external links to alternative dietary approaches
**Module 6: Practical tips**	**Supermarket tour**	Animation virtually walking the user around a supermarket providing practical tips on what to look out for when shopping and resisting temptation
	**Understanding food labels**	How to facilitate weight loss by understanding food labels
	**Portion sizes**	Recommended portion sizes of different food groups to promote weight loss and maintenance
	**Healthy eating recipes**	Provides external links to healthy eating recipes to promote weight loss and maintenance
**Module 7: Physical activity, exercise and NAFLD**	**How changing lifestyles contribute to NAFLD and weight gain**	Animation communicating how reduced physical activity levels are linked to weight gain and NAFLD
	**Combining physical activity and diet for weight loss**	The benefits of combining physical activity and dietary change to promote weight loss
	**Physical activity, exercise and NAFLD**	The difference between physical activity and exercise; how physical activity and exercise can benefit the liver and lead to other health benefits
	**How much physical activity do I need to do?**	The required amount, intensity and type of physical activity and exercise required to manage NAFLD; ideas for increasing physical activity and exercise levels
**Module 8: Steps to Success**	**Tips for setting your own goals**	Tips for setting SMART goals
	**Goal setting**	Tool to facilitate personalised behavioural goal setting for diet and physical activity
	**Set a food goal**	Tool to facilitate monitoring of diet against dietary goals
	**Step tracking**	Tool to facilitate monitoring of daily step counts and track progress
	**Weight tracking**	Tool to facilitate weight monitoring and track progress
	**Maximising chances of success**	Animation prompting use of SMART goals; self-monitoring and support from family and friends to maximise the chances of success

### VITALISE lifestyle coaching

As part of the VITALISE intervention, patients will have access to monthly personalised tele-coaching sessions delivered by trained lifestyle coaches. Coaches will have received training on what NAFLD is, how it is investigated, different treatment options (aligned with clinical guidelines) and the evidence supporting lifestyle intervention for the management of NAFLD in accordance with clinical guidelines. Coaches are also trained in the use of brief motivational techniques and will have an in-depth knowledge of the information content and self-regulation tools provided to enable signposting of patients during coaching sessions.

The initial coaching appointment will last approximately 20 min and subsequent appointments 10–15 min. Coaching duration and frequency were informed by findings from previously studies [15, 22]. The coaches will facilitate person-centred behavioural goal setting and encourage self-monitoring of lifestyle behaviours by using the tracking tools embedded within VITALISE.

### Additional support

A member of the research team will telephone each patient at the end of 12 weeks to answer any questions, promote continued engagement, and record any adverse events. Patients will be asked to self-report their weight, physical activity levels (step count and questionnaire) and complete the PAM.

Email reminders will be sent out to each patient once each week for the first four weeks, then monthly thereafter to prompt continued engagement with the intervention. Patients will have the opportunity to contact a member of the research team via telephone or email for assistance if they experience any technical difficulties during the study.

### Outcomes and criteria for success

Criteria to judge the feasibility of progressing to a larger scale evaluation:

#### Recruitment rate

Six patients recruited per month over a 6-month time period.

#### Intervention uptake and engagement

 ≥ 80% of patients recruited logging in to the intervention.

#### Completion and follow-up rate

 ≥ 70% of those accessing the intervention providing data at 6-months follow-up.

These criteria aim to retain a sufficient sample size to determine acceptability and feasibility.

#### Primary outcome measures

In line with NIHR guidance on feasibility studies, the focus will be to estimate important parameters that are needed to inform the design of a subsequent larger evaluation, if appropriate. Table 3 details the feasibility study parameters.

**Table 3:** Feasibility study parameters

Parameter	Method of assessment
Recruitment rate	The number of eligible patients invited, who provide informed consent to participate in the study, versus those who decline, with reason(s) recorded
Intervention uptake, engagement and adherence	The number of patients who login to access VITALISE The mean and median number of times VITALISE is accessed over the 6-month time period
Completion and follow-up rate	The number of patients who complete the intervention, baseline and follow-up assessment outcome measures. Reasons for loss to follow-up will also be recorded

We will collect demographic data (age, sex, ethnicity, socio-economic status) on patients who decline to take part and record reasons for declining/unsuitability to inform the way in which the programme is promoted in the future.

Acceptability of VITALISE will be assessed qualitatively using in-depth, semi-structured interviews and all patients recruited will be invited to take part. Topics will focus on patients’ perceived expectations, benefits, motivations, and barriers to using/accessing VITALISE. These topics will be explored in relation to age and liver disease severity. We will qualitatively assess fidelity of receipt and enactment of the intervention to provide an indication of whether participants used the intervention as intended to make behavioural changes. Our interview topic guide will contain specific questions about each component of the intervention to determine whether they were received by participants and whether they utilised each component as intended. This information will inform optimisation of the intervention ahead of a large-scale evaluation, if appropriate. Those who drop out of the intervention will be invited to interview to explore reasons for drop-out. Findings will inform intervention optimisation.

We will also collect data on the number of patients who log in to the intervention, the number of times they access each module, attend coaching appointments, and the length of coaching calls, to enable us to provide context around study outcomes – i.e., is it likely that any preliminary changes observed are a consequence of engagement with the intervention.

### Secondary outcome measures

Body weight, height, waist and hip circumference, blood pressure, blood tests (glucose, lipid profile, LBTs, FBC, HbA1c), liver stiffness measurement and calculation of clinical scores (QRISK^3^, FIB-4, NFS) will be measured as part of standard care.

#### Physical activity

Physical activity will be objectively measured using an ActiGraph GT9X accelerometer worn on the non-dominant wrist for a minimum of 8 h per day over seven consecutive days during the first and last week of the intervention. Daily total activity counts, steps, time spent sedentary, and time spent in light, moderate, and vigorous activity will be recorded. Freedson thresholds based on metabolic equivalents will be used to demarcate to intensity of physical activity: sedentary (≤ 100 counts/min), light (101–1951 counts/min), moderate (1952–5724 counts/min), and vigorous activity (≥ 5725 counts/min) [23, 24]. Self-reported physical activity will be assessed with the Godin Leisure-Time Exercise Questionnaire using the total leisure activity score [20] at baseline, 12-weeks and 6-months.

#### Patient activation/empowerment

Patients will be asked to complete Patient Activation Measure (PAM) [21] at baseline, 12-weeks and 6-months. The PAM questionnaire collects data on an individual’s knowledge, skills and confidence to manage their health and is a global measure of an individual’s overall ability to manage their health.

Questionnaires will be given to patients in the clinic to take home and return by post in pre-paid envelopes alongside their accelerometer. We will look at return rates for questionnaires/accelerometers as part of the feasibility study and address issues/seek patient feedback on ways to improve this for the larger scale evaluation as necessary.

### Safety reporting

This is a small-scale feasibility study, and therefore a formal data monitoring committee will not be convened. We do not anticipate any major risks or adverse events to arise as a result of taking part in this study. If an adverse event does occur, it will be recorded and categorised in terms of relatedness and severity in line with the CTCAE criteria. The research team will meet bi-weekly to monitor interim safety/feasibility data by reviewing recruitment/adherence rates and adverse events. The NuTH NHS Trust will be informed according to trust policy.

### Data and statistical analysis

#### Quantitative analysis

The flow of participants throughout the trial will be reported in a Consolidated Standards of Reporting Trials (CONSORT) flowchart. Reasons for declining to take part in and for withdrawing from the study will also be recorded in the CONSORT flowchart. Descriptive statistics will be used to present baseline characteristics and feasibility outcomes. A paired t-test or Wilcoxon signed-rank test (depending on data distribution) will be used to evaluate changes in outcomes from pre- to post-intervention, with the mean difference and 95% confidence interval presented.

#### Qualitative analysis

Interviews will be audio recorded, transcribed verbatim, and analysed using thematic analysis [25]. All transcripts will be coded independently by one member of the research team, with a proportion (~ 50%) independently coded by a second research team member. Codes and potential themes will be discussed and agreed at the research team level and agreed for reporting purposes.

### Research ethics approval

An independent NHS Research Ethics Committee (North East—Tyne & Wear South Research Ethics Committee) has provided a favourable ethical opinion for this study to commence (IRAS ID: 313,662).

### Patient and public involvement

We have consulted extensively with patients with NAFLD to identify their supported self-management needs and preferences. A consistent finding is their lack of knowledge about what NAFLD is, how it progresses (and that it can progress) and current management options (“*the only thing they said was to try and sort of lose a bit of weight…apart from that I’ve never ever had any advice or anything else”*). Most patients we consulted did not understand the links between their lifestyle behaviours and NAFLD, including its progression. A lack of knowledge that NAFLD can be managed by making lifestyle changes was evident. This is likely due to the lack of NAFLD-specific resources available to facilitate communication with patients (“*I couldn’t really go into it. It was so brief, what I got off my GP. She did tell me I could Google it and read up about it…But I haven’t*”).

The findings from a series of interviews and workshops with patients have led to the development of VITALISE. The involvement of specifically trained lifestyle coaches was considered an essential intervention component to promote ongoing engagement and help patients overcome barriers to achieving clinically and personally meaningful goals.

Patients who assessed usability of VITALISE reported feeling empowered, informed and motivated to make lifestyle changes.

### Modification of the protocol

Any modifications to the protocol will be agreed by the research team and study sponsor, and approved by an independent NHS Research Ethics Committee.

## Discussion

Lifestyle intervention is recommended by national and international clinical guidelines for the management of NAFLD, regardless of disease severity [8–10]. However, clinicians struggle to support patients with NAFLD to make and sustain lifestyle changes due to a lack of NAFLD-specific interventions and tools to use in clinical practice. People with NAFLD find weight loss and weight loss maintenance a significant challenge and report a lack of NAFLD-specific, tailored support from HCPs [11, 12].

VITALISE is an evidence-informed intervention comprising of structured education and self-regulation tools to help support changes in diet and physical activity behaviours to initiate and maintain weight loss. VITALISE provides credible information for patients relating to what NAFLD is (and isn’t), and how it links to their lifestyle behaviours.

VITALISE offers patients the opportunity to access tailored dietary and physical activity support in their own time, outside of the hospital setting. This may remove some of the documented barriers to participation if face-to-face attendance at appointments is not required. Despite its potential, there are uncertainties relating to the feasibility and acceptability of using digital interventions to support clinical care in people living with NAFLD.

This mixed-methods study will address uncertainties relating to the feasibility and acceptability of delivering online, behaviour change interventions to patients with NAFLD. The findings will inform optimisation of the intervention, if required, and the assessment of outcomes will provide information on the likely size and variability of intervention effects. Collectively, the data generated will inform the design of a subsequent, adequately-powered, larger scale evaluation of VITALISE, if appropriate.

## Data Availability

This manuscript does not contain any data. Supporting material is available as supplementary information files.

## Abbreviations

NAFLD: Non-alcoholic fatty liver disease
VITALISE: Inter**V**ention to promote l**I**fes**T**yle change in non-**A**lcoholic fatty **LI**ver disea**SE**
NASH: Non-alcoholic steatohepatitis
HCP: Healthcare professional
NuTH: Newcastle upon Tyne Hospitals NHS Foundation Trust
PAM: Patient activation measure
LBT: Liver blood tests
FBC: Full blood count
LSM: Liver stiffness measurement
FIB-4: Fibrosis-4 score
NFS: NAFLD fibrosis score
T2DM: Type 2 diabetes
CONSORT: Consolidated standards of reporting trials
